# Hemobilia in a Child Due to Right Hepatic Artery Pseudoaneurysm: Multidetector-row Computed Tomography Demonstration

**DOI:** 10.4103/1319-3767.77250

**Published:** 2011

**Authors:** Nisar A. Wani, Tariq A. Gojwari, Naseer A. Khan, Tasleem L. Kosar

**Affiliations:** Department of Radiodiagnosis and Imaging, Sher-I-Kashmir Institute of Medical Sciences (SKIMS), Srinagar, J & K, India

**Keywords:** Hemobilia, hepatic artery pseudoaneurysm, Multidetector-row computed tomography

## Abstract

We present a case of a 12-year-old boy who developed upper gastrointestinal bleeding in the form of hematemesis and melena 1 month after blunt trauma to liver. Computed tomography (CT) angiography with multidetector-row CT demonstrated pseudoaneurysm of right hepatic artery related to old liver laceration to be the cause of the bleeding. Pseudoaneurysm was resected using the roadmap provided by CT angiography findings.

Hemobilia is a rare cause of gastrointestinal (GI) bleeding that develops as a result of communication between blood vessels and the biliary tract. It should be considered in cases of hematemesis and melena when no GI lesion is seen on several endoscopies in the presence of a suggestive clinical context and symptoms from the biliary tract.[1,2] Most cases of hemobilia are the result of accidental liver trauma or iatrogenic injuries resulting from invasive diagnostic procedures involving the liver. The mean time period between trauma and hemobilia is 4 weeks.[2] We report here the case of a child who developed hemobilia 1 month after blunt trauma to the abdomen. Computed tomography (CT) angiography revealed a pseudoaneurysm of the right hepatic artery as the cause of hemobilia.

## CASE REPORT

A 12-year-old boy presented with a history of hematemesis and melena from 10 days. The child had blunt abdominal trauma due to road traffic accident 1 month earlier for which he was operated at an outside hospital for hemoperitoneum and at laparatomy a large laceration of right lobe of liver was seen. Hemostasis was achieved by packing, which was confirmed on re-look laparotomy on the 2^nd^ day, and a drain was kept in the perihepatic space; no attempt for primary repair of liver laceration was done. The child stabilized hemodynamically and was discharged 10 days after surgery; his parents were advised to monitor the color and volume of the drained fluid. After surgery, although asymptomatic, he continued to drain some 100–200 mL of blood containing fluid from the drain daily and then developed GI bleeding.

On examination at presentation with GI bleed, the child was pale but afebrile; scleral conjunctiva showed icterus. His pulse was 108 beats/min and blood pressure on admission was 90/50 mmHg. Liver and spleen were not palpable. Hemoglobin was 6 g/dL, alkaline phosphatase was 400 IU/L, gamma-glutamyltransferase was 180 IU/L, serum bilirubin was 4 mg/dL, and aminotransferases were slightly elevated. Platelet count, prothrombin time, partial thromboplastin time, electrolytes, blood urea nitrogen, creatinine, and amylase were all normal.

The child continued with vomiting of blood and passage of black tarry stools. Upper GI endoscopy was performed after blood transfusion and showed bulky clots in the stomach and fresh blood in the duodenum; no varices or mass lesion were seen in the esophagus or stomach. The major duodenal papilla was not examined satisfactorily because of the presence of a lot of blood. Abdominal ultrasonography (US) revealed a septate fluid collection around the right lobe of the liver; intrahepatic and extrahepatic bile ducts were slightly dilated, no calculus or worm or debris was seen in the ducts.

**Figure 1:**
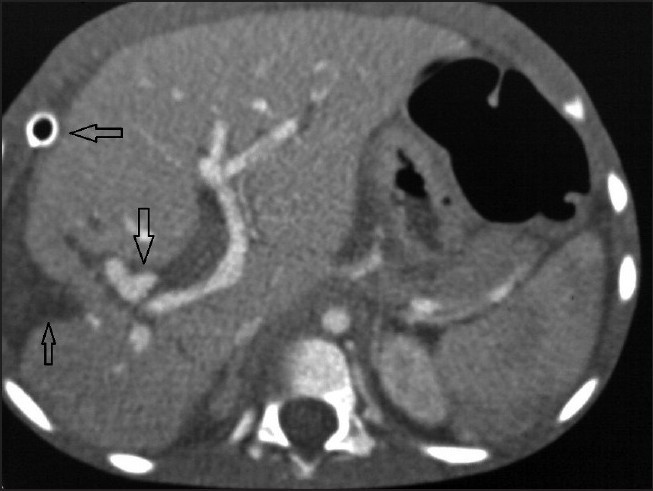
Axial contrast-enhanced CT image showing defect in the right lobe of the liver with adjacent perihepatic fluid collection (upward arrow). Enhancing lesion is seen anterior to the right branch of the portal vein (down ward arrow). Drain tip is seen anteriorly, in perihepatic location (leftward arrow)

**Figure 2:**
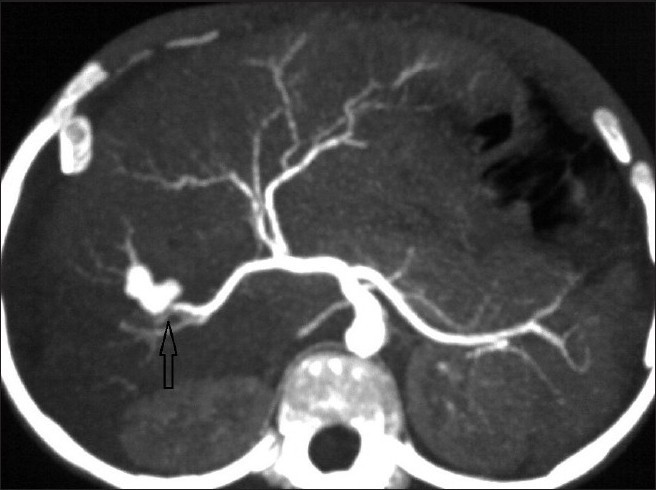
Axial plane thick multiplanar reformation CT angiography image showing pseudoaneurysm of the right branch of the hepatic artery (upward arrow)

**Figure 3:**
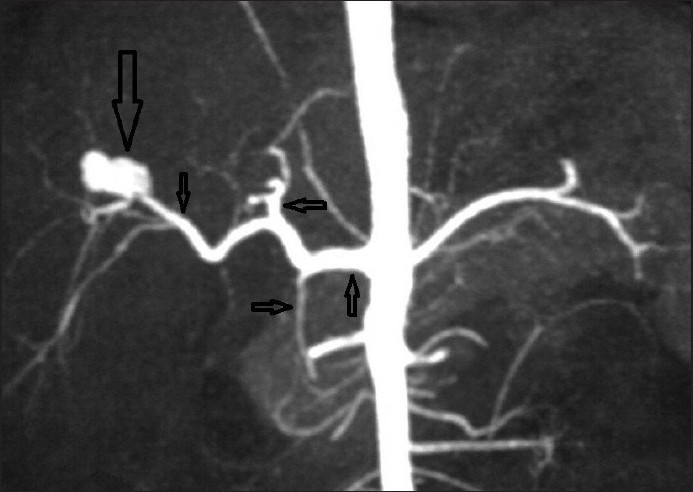
Coronal plane thick multiplanar reformation CT angiography image showing right hepatic artery pseudoaneurysm with normal common hepatic artery (upward arrow), left hepatic artery (leftward arrow), and gastroduodenal artery (rightward arrow); proximal right hepatic artery is normal (small downward arrow) with pseudoaneurysm arising further distally (large downward arrow)

Contrast-enhanced CT (CECT) study of abdomen was performed with a 64-slice multidetector-row-CT (MDCT), which revealed a defect in the right lobe of the liver with adjacent perihepatic fluid collection with drain tip more anteriorly; enhancing lesion was seen anterior to the right branch of the portal vein [Figure 1]. CT angiography reconstructed as three-dimensional (3D) volume-rendered and thick multiplanar reformation (MPR) images from the arterial phase of CECT revealed the diagnosis [Figures 2 and 3]. An irregular shaped structure showing same enhancement as adjacent celiac artery and aorta was seen deep to the old laceration in the right lobe, arising from the right branch of the hepatic artery suggestive of pseudoaneurysm [Figures 2 and 3]. Celiotomy was planned for the treatment as embolization was not available. Surgical resection of pseudoaneurysm was done. The patient tolerated the surgery well and made an uneventful recovery; his liver function tests gradually returned to normal. Hematemesis and melena subsided after surgery. He was discharged on the 10th postoperative day and was seen doing well in the follow-up 3 months after operation.

## DISCUSSION

Hemobilia results from a communication between the hepatic arterial and biliary systems and is quite an unusual complication of accidental liver injury. Other causes of hemobilia are iatrogenic liver trauma from liver biopsy, transhepatic cholangiography and biliary drainage, and inflammatory, tumoral, vascular disorders, and laparoscopic cholecystectomy.[3,4] Hemobilia develops in 2.5% of patients after accidental liver trauma and in 3%–7% of patients after iatrogenic liver trauma.[3] Most common underlying cause of posttraumatic hemobilia is a pseudoaneurysm. Bile leak and superimposed infection are the contributing factors suggested for the development of posttraumatic hepatic artery pseudoaneurysm.[4,5] Prolonged drain of fluid following a deep parenchymal liver laceration in our case was likely due to bile leak as was also suggested by increased serum bilirubin. Initial surgical intervention and a drain close to the site of injury kept in place for an extended duration of time were the predispositions to infection. The mean time period between traumatic injury and hemobilia is reported to be around 4 weeks; in the case of percutaneous liver biopsy, the mean onset time is around 7 days. Nevertheless hemobilia may occur many months after trauma. A classic triad of hemobilia (biliary colic, obstructive jaundice, and intestinal bleeding) is absent in almost 70% of cases making clinical diagnosis difficult.[1,2,5] However, the history of hematemesis with melena after abdominal trauma in the presence of abnormal liver function tests may suggest hemobilia.[1,3]

Diagnostic procedures include upper GI endoscopy, which besides excluding other causes of bleeding may reveal blood flowing from the major duodenal papilla thus facilitating recognition of hemobilia. Abdominal US and CT may reveal dilatation of bile ducts with blood within the ducts and the gall bladder. Hemobilia may cause a CT scan finding of mixed or uniform high-attenuation blood within the gallbladder lumen. High-density material within the lumen of the gallbladder on CT scan, however, is also seen with gallstones, vicarious excretion of intravenous contrast, biliary sludge, and milk of calcium bile.[3,5–7] These entities need to be considered in the differential diagnosis of hemobilia on CT. CT has undergone considerable improvement over the past several years with the introduction of MDCT, including rapid scanning in the arterial phase of enhancement; a 64-slice MDCT allows submillimeter imaging and the creation of isotropic data sets. This, along with significant advancements in 3D imaging software in the form of volume rendering, MPR, and maximum intensity projection algorithms now make it possible to obtain high-resolution images of the abdominal aorta and its splanchnic branches.[8] In the past, patients with hemobilia would need conventional angiography to look for a suspected vascular abnormality, such as pseudoaneurysm. However, today CT can be used initially as a primary vascular imaging technique for hemobilia evaluation. CT angiography using MDCT is fast replacing catheter arteriography for diagnosis of pseudoaneurysms and the latter is now used for therapeutic procedure guidance only.[8]

Classic treatment options for hepatic artery pseudoaneurysms include surgical resection of the pseudoaneurysm and revascularization of the injured vessel segment, ligation of the hepatic artery, or stent graft interposition.[9,10] CT-guided direct percutaneous puncture of the aneurysm lumen and injection of thrombin is described; however, catheter-based endovascular approaches, including radiologic coil embolization are presently the most favored treatment.[3–6,9,10] The size, morphology, and location of the aneurysm as determined by CT angiography can help guide treatment. For example, intrahepatic aneuryms can be embolized with coils, microspheres, or glue. At the level of the common hepatic artery, embolization or surgical ligation can be performed; if the aneurysm involves the origin of the gastroduodenal artery, surgery may be easier than a percutaneous approach. Saccular aneurysms with a good neck can be treated with embolization. Saccular or fusiform aneurysms without a good neck require surgical intervention.[6,11] Thus preoperative diagnosis of hepatic artery pseudoaneurysm is noninvasively made with CT angiography, which facilitates its management. Doppler sonography has also been used for diagnosis and is preferred for follow-up of the patients after endovascular or surgical treatment of hepatic artery pseudoaneurysm.[11]

In conclusion, GI bleeding after traumatic liver injury in children should give rise to a clinical suspicion of hemobilia and the underlying hepatic artery pseudoaneurysm. CT angiography should be performed for early diagnosis and management in such patients.
